# Modeling Routes of Chronic Wasting Disease Transmission: Environmental Prion Persistence Promotes Deer Population Decline and Extinction

**DOI:** 10.1371/journal.pone.0019896

**Published:** 2011-05-13

**Authors:** Emily S. Almberg, Paul C. Cross, Christopher J. Johnson, Dennis M. Heisey, Bryan J. Richards

**Affiliations:** 1 Northern Rocky Mountain Science Center, United States Geological Survey, Bozeman, Montana, United States of America; 2 The Huck Institutes of the Life Sciences, Pennsylvania State University, University Park, Pennsylvania, United States of America; 3 Prion Research Laboratory, National Wildlife Health Center, United States Geological Survey, Madison, Wisconsin, United States of America; 4 National Wildlife Health Center, United States Geological Survey, Madison, Wisconsin, United States of America; Albert Einstein College of Medicine, United States of America

## Abstract

Chronic wasting disease (CWD) is a fatal disease of deer, elk, and moose transmitted through direct, animal-to-animal contact, and indirectly, via environmental contamination. Considerable attention has been paid to modeling direct transmission, but despite the fact that CWD prions can remain infectious in the environment for years, relatively little information exists about the potential effects of indirect transmission on CWD dynamics. In the present study, we use simulation models to demonstrate how indirect transmission and the duration of environmental prion persistence may affect epidemics of CWD and populations of North American deer. Existing data from Colorado, Wyoming, and Wisconsin's CWD epidemics were used to define plausible short-term outcomes and associated parameter spaces. Resulting long-term outcomes range from relatively low disease prevalence and limited host-population decline to host-population collapse and extinction. Our models suggest that disease prevalence and the severity of population decline is driven by the duration that prions remain infectious in the environment. Despite relatively low epidemic growth rates, the basic reproductive number, *R*_0_, may be much larger than expected under the direct-transmission paradigm because the infectious period can vastly exceed the host's life span. High prion persistence is expected to lead to an increasing environmental pool of prions during the early phases (i.e. approximately during the first 50 years) of the epidemic. As a consequence, over this period of time, disease dynamics will become more heavily influenced by indirect transmission, which may explain some of the observed regional differences in age and sex-specific disease patterns. This suggests management interventions, such as culling or vaccination, will become increasingly less effective as CWD epidemics progress.

## Introduction

The intuition of many researchers and managers about disease dynamics is based on a few well-studied empirical and theoretical disease systems [1]. These systems tend to involve a single host species with direct subject-to-subject transmission. However, many host-pathogen systems are multi-host and multi-pathogen with direct and indirect modes of transmission [2]. Previous studies have found that indirect transmission improves the probability of pathogen invasion and persistence [3], [4], [5]. These studies have typically focused on pathogens whose lifespans in the environment are much shorter than that of their hosts. A number of pathogens exist, however, where the duration of infectiousness of the disease agent in the environment can greatly exceed the typical host's lifespan. Chronic wasting disease (CWD), a transmissible spongiform encephalopathy (TSE) or prion disease, of cervids, is one such persistent pathogen. Similar to the other TSEs, including sheep scrapie, bovine spongiform encephalopathy (BSE; “mad cow” disease) and Creutzfeldt-Jakob disease (CJD) in humans, CWD is caused by an infectious, aberrant form of the prion protein, referred to as a “prion.” During the course of infection, CWD prions accumulate in lymphoid tissue and the central nervous system and, although the mechanism of pathogenesis is unknown, accumulation of CWD prions in the body coincides with tissue damage and eventual host death [6], [7]. Disease progresses very slowly in deer, usually taking in excess of a year for clinical signs to develop, much of the time during which the animal is infectious [8], [9], [10]. Although originally assumed to be primarily directly transmitted, CWD has been shown to be both directly and indirectly transmitted [11], [12], [13], through contact with saliva [14], [15], urine [15], feces [10], [16], infected carcasses and prion-contaminated environments [17], [18].

Chronic wasting disease affects North American mule deer (*Odocoileus hemionus*), white-tailed deer (*Odocoileus virginianus*), elk (*Cervus elaphus*), and moose (*Alces alces*). The origin of CWD is unknown, but following its discovery among a captive population of mule deer in 1967, it has since been found to infect several wild cervid populations throughout North America (for map of current distribution, see: http://www.nwhc.usgs.gov/images/cwd/cwd_map.jpg). The largest outbreaks, in terms of numbers of animals infected, are occurring among elk, mule deer, and white-tailed deer in northern Colorado and southeastern Wyoming, and among white-tailed deer in southern Wisconsin and northern Illinois. Intensive surveillance efforts in both regions have yielded estimates of apparent prevalence (the number infected: total sampled), distinct age and sex-specific infection hazards [19], and host population trends over time. In Wyoming's endemic region, the prevalence of CWD among mule deer has grown from ∼11% to ∼36% from 1997–2007, with local annual prevalence growth rates in excess of 1.15 (calculated based on an exponential regression fit to Wyoming Game and Fish data [20], [21]). Apparent age and sex-specific prevalences vary somewhat by species and locale, but in general, males exhibit higher prevalences than females (although the opposite has been found in a Wyoming white-tailed deer herd [22]), and males appear to exhibit an age-prevalence peak at 5–6 years old whereas the pattern among females is less clear [23], [24]. In northcentral Colorado, biologists have estimated local annual prevalence growth rates to be as high as 1.2–1.25 among male mule deer [24]. The presumably younger Wisconsin epidemic has yielded CWD prevalences of 5–15%, with annual increases of 1.08 now detectable [19], and with middle-aged (1–3 yr olds) animals and males experiencing higher apparent rates of infection than very young, old, or female deer [19], [25]. In several high-prevalence study areas in Wyoming and Colorado, biologists have witnessed coincident population declines of up to 30–50% [21], [26].

The majority of CWD modeling studies to date have focused on direct transmission and how subject-to-subject contacts relate to host density [27], [28], [29]. Under the direct transmission paradigm, the apparently slow development of CWD in Wisconsin and Wyoming/Colorado over the last 15–20 years implies that the *R*_0_ of CWD may be relatively low, and that as a result, even limited control measures may be effective. However, despite culling programs aimed at reducing deer densities, and hence transmission rates, CWD continues a stochastic increase in prevalence in Wisconsin [30] and Colorado [31]. Experiments with penned deer demonstrate that indirect environmental transmission occurs [11], [12] and associated modeling studies indicate environmental persistence of CWD is likely affecting disease dynamics [32]. Soil is a likely environmental reservoir for CWD infectivity [17], [18] and while the duration of prion persistence in the environment is unknown, laboratory work to date suggests that prions in soil are extremely stable with only limited degradation [33], [34]. Prions bind to soil particles, and binding to clays and some soils increases both prion bio-availability and infectivity [35], [36], [37]. Pen studies with CWD prions suggest infectivity persists for at least 2 years [11], and studies of the closely related prion disease, sheep scrapie, have shown that scrapie prions can remain infectious and bio-available in the environment for at least 16 years [38].

We hypothesized that long-term environmental prion persistence would dramatically alter deer-CWD dynamics as well as the potential for host-pathogen co-existence. In particular, we hypothesized that even though the annual prevalence growth rates of CWD may be relatively low, the pathogen's basic reproductive number (the average number of secondary infections caused by an index case in a completely susceptible population), *R*_0_, may be very high [39]. This is because unlike in the case of direct transmission, the persistence of an environmental reservoir allows an infected host to cause secondary infections long after its death. Estimating the basic reproductive number of CWD as well as the degree to which transmission is indirect, is critical to understanding the level and type of effort needed for disease control and eradication.

Using simulation models, we explore the impacts of indirect transmission on CWD dynamics among cervids. Given uncertainty surrounding the details of CWD transmission, we model dynamics assuming a wide range of direct and indirect transmission rates, functional forms of transmission, and prion degradation rates. In addition, we phenomenologically account for the possibility that individuals and/or prions are spatially clumped or aggregated using a non-spatial model. Outputs from our model include time-series of prevalence, host population size, and the dynamics of the environmental reservoir. We also calculate the basic reproductive number, *R*_0_, for plausible simulation outcomes and demonstrate that environmental prion persistence is a critical factor in the timing and extent of effective control efforts.

## Methods

### Model structure

We developed a discrete, aspatial, susceptible(*S*)-exposed(*E*)-infectious(*I*)-clinical(*C*) model to explore the dynamics of CWD in a population of 15,000 North American mule deer. The model did not include sex or age structure; instead, we used the weighted geometric averages of empirically measured age and sex-specific mule deer survival and birthing rates as density-independent per capita probabilities of survival, *s*, and of giving birth, *α* [29], [40]. Reproduction took place as a single birth pulse at the end of the year and was calculated as the product of the density-dependent, per capita probability of reproducing [41], [42], [43], [44]

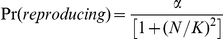
where *N* is the population size and *K* is the carrying capacity of deer (20,000), and the average number of offspring per birth.

**Figure 1:**
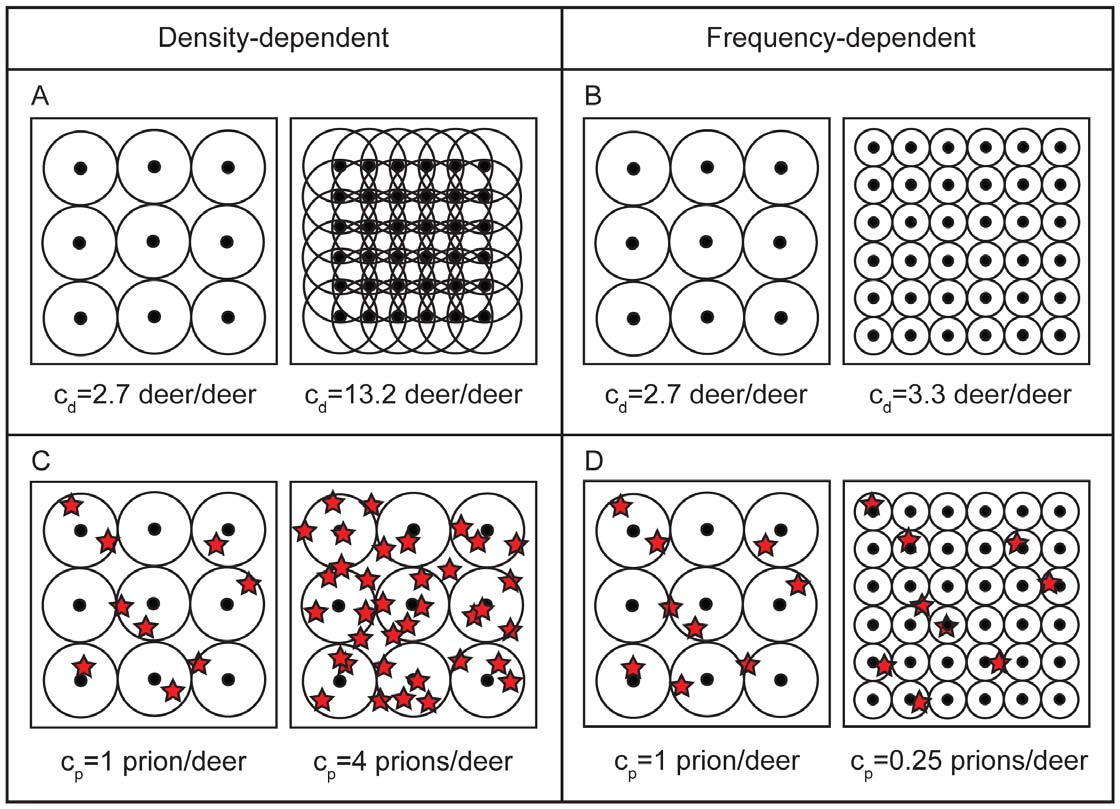
Schematic of density (DD) and frequency (FD) dependent transmission applied to direct and indirect transmission. Dots and circles represent deer and their home ranges, and red stars represent infectious prions in the environment. Deer and prion contacts (means denoted by and c_d_ and c_p_) are defined by shared or overlapping home range edges, and home range overlap with prions, respectively. A) Under DD direct transmission, increasing host density (by a factor of 4) is assumed to increase host contact rates. B) Under FD direct transmission, spatial or social structuring keeps contact rates largely independent of host density (the slight increase displayed is due to edge effects). C) Under DD indirect transmission, increasing prion density (by a factor of 4) increases prion contacts. D) Under FD indirect transmission, prion contacts scale with host density; as host density increases, spatial structuring reduces home range size and hence per capita rates of prion contact.

We modeled transmission to include both direct and indirect transmission over a range of functional forms and aggregation. We temporarily ignore births and deaths as we develop our transmission model below. Let *Z* and *S* be the infectious and susceptible counts, respectively, where *Z* = *I*+*C* and *N* = *S*+*E*+*I*+*C*. Let the environmental concentration of prions be *V*. The force-of-infection, *λ*, is the instantaneous per capita infection rate, and can be modeled as *λ* = *β_d_Z*+*β_i_V* where *β_d_* and *β_i_* are, respectively, the direct and indirect transmission coefficients. Such a model is appropriate for systems where the direct and indirect transmission rates are dependent on the density of hosts and the concentration of prions in the environment, respectively. This model generalizes the classical concept of density-dependent transmission to both direct and indirect transmission. An alternative model, which similarly generalizes the classic concept of frequency-dependence, is *λ* = (*β_d_Z*+*β_i_V* )/*N*. Such a model is appropriate if spatial partitioning, such as social grouping or rigid territories, makes the direct contact rate independent of total population density. However, under this model formulation, the indirect contact rate becomes dependent on total population density. We suggest that although contact rates with the environment might be fixed, the proportion of contaminated environment encountered may be inversely related to total host density. As *N* increases, individual home ranges may decrease in size [45], [46], reducing the proportion of the landscape encountered on a per capita basis. As a result environmental transmission rates may decrease with increasing deer numbers for a given level of prion contamination (Figure 1).

**Figure 2:**
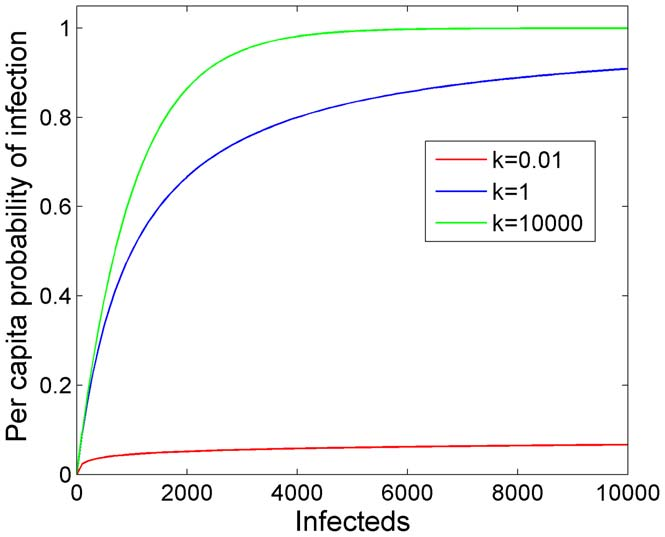
Aggregation and the probability of infection. Aggregation reduces the rate at which additional infectious individuals or particles contribute to the probability of infection. High aggregation is represented by *k* = 0.01, where as low aggregation is represented by *k* = 10000. Simulation run assuming density-dependent (*ε* = 0) direct transmission with *β_d_* = 0.001.

In this study, we assumed roughly the same density- or frequency-dependent processes are occurring with respect to both direct and indirect transmission. In reality, some intermediate mechanism between pure density- and frequency-dependence is most likely, motivating us to work with a more general model
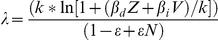
for *ε* in [0,1] and *k* in (0,10000]. When *ε* equals 0 the model is density dependent, and when *ε* equals 1 the model is frequency dependent. The parameter *k* adjusts the relative importance of each additional infectious deer or prion (Figure 2). As *k* approaches infinity all infectious individuals and prions are equal; as *k* approaches zero each added infectious individual or prion diminishes the incremental per-individual contribution. This model phenomenologically accounts for heterogeneity of infection risk [47], [48]. By varying just two parameters, *ε* and *k*, a rich variety of potential mechanisms can be produced. High aggregation (i.e. *k* = 0.01) translates to an infection rate that is relatively constant over a large range in infectious individuals and/or prions (Figure 2). This results in similar dynamics to frequency dependence transmission. Therefore, we only modeled frequency-dependent transmission in the absence of aggregation. We converted the instantaneous rate (hazard or intensity) *λ* to a finite unit time infection probability as 1−exp(−*λ*).

Simulations were initiated by introducing one infectious individual into a population of 15,000 mule deer, the approximate size of several managed populations in Colorado and Wyoming [49]. We adopted a multi-compartmental ‘box-car’ approach [50] to create exposed, infectious, and clinical periods that were roughly log-normally distributed [51], [52], [53]. Upon infection, individuals transitioned from the susceptible class into a series of 35 exposed classes (mean duration of exposure = 27 weeks, range = 15–35 weeks), 44 infectious classes (mean duration of infectious phase = 36 weeks, range = 25–44 weeks), and 36 clinical classes (mean duration of clinical phase = 17 weeks, range = 1–36 weeks), followed by death (Figure S1 & S2, Table S1) [8], [9], [10]. The model operated on a weekly timestep and we ordered events as follows: transmission, transition between disease states *S*, *E*, *I*, and *C*, host survival, shedding of prions, and prion ‘survival.’ While in the infectious and clinical phases, individuals were assumed to regularly shed prions and to contribute to the environmental load of prions upon their death. Individuals experienced elevated disease-associated mortality only during the clinical phase (Figure S1, Table S1). We use the term ‘prion survival’ to refer to the rate at which prions remain bio-available and infectious on the landscape. We ran simulations over a range of prion survival rates, γ (weekly survival probabilities: 0.9481, 0.9868, 0.9967, 0.9978, 0.9983, translating to half-lives of 0.25, 1, 4, 6, and 8 years, respectively). For each unique combination of direct and indirect transmission rates, prion survival, and functional form of transmission, we ran 10 replicate simulations for 200 years. The model was stochastic in that binomial trials governed the random outcomes of host survival and reproduction, transmission, movement between disease classes, and prion survival.

### Parameterization

We calculated the weighted geometric averages of age and sex-specific birthing (α = 0.55, annually) and survival probabilities (*s* = 0.991, weekly), as well as the average number of offspring per birth (1.7), from empirical datasets on mule deer [29], [40], [49], [54]. Our decision to model density-dependence as operating on the per-capita probability of giving birth should capture density-related changes to the age of first reproduction, doe conception/birth rates, or fawn survival through the first winter [41], [42], [43], [44]. These distinctions are probably irrelevant for modeling CWD dynamics as fawns that die early are unlikely to contribute to disease transmission. Our model yields a maximum annual finite growth rate of 1.30, a value very similar to the upper range estimated for wild mule deer (1.16–1.27 [55]), and thus we consider it an adequate approximation.

Disease parameters were fitted to data collected from pen studies of CWD in deer [8], [9], [10]. Average prion survival rates are unknown, however pen studies have shown that CWD prions can remain bio-available in the environment for at least 2 years [11], and studies of the closely related disease, sheep scrapie, have shown that scrapie prions can remain infectious for at least 16 years [38]. Hence, we chose a wide range of prion survival values over which to simulate.

The two components of transmission, contact rates between infectious and susceptible individuals and the probability of infection given contact, are unknown. Furthermore, the infectious dose required for natural infection is unknown. For computational ease, we assumed that individuals shed 2 prion units per timestep while in the infectious and clinical phases. In our model, animals released between 52 and 160 (mean = 106) infectious prion units over their infectious lifetime and 100 prion units upon death. We based these values upon reports of the infectious titers of deer excrement and saliva [10], [15], abnormal prion protein throughout the lymphatic system [56] and high titers of infectivity in the nervous system [57].

For simplicity, and in the absence of detailed empirical data, we assumed the shedding rate or the prion load at death was not dependent on the time since infection, and we assumed that exposed (state *E*) individuals contributed no prions at death. The units on *V*, the concentration of prions in the environment, are essentially dimensionless, and only the product *β_i_V* is relevant in the model, which was scaled empirically to produce plausible outcomes. We initially ran simulations over a wide range of direct and indirect transmission rates and combinations thereof, and then visually inspected the output and narrowed our ranges of β*_d_* and β*_i_* to capture the relevant parameter space. Final simulations reflect 30–66 combinations of β*_d_* and β*_i_* per functional form of transmission selected to cover the widest range of potential outcomes from low to high prevalence (Table 1).

**Table 1:** Direct (*β_d_*) and indirect (*β_i_*) transmission coefficients employed in model simulations.

Functional form of transmission	k	γ = 0 (Direct only)	γ = 0.9481 (HL = 0.25 yrs)	γ = 0.9868 (HL = 1 yr)	γ = 0.9967 (HL = 4 yrs)	γ = 0.9978 (HL = 6 yrs)	γ = 0.9983 (HL = 8 yrs)
Density-dependent (ε = 0)	0.01, 1, 10000	*β_i_* = NA	*β_i_* = [3e-8,1.8e-7], n = 5; [1.1e-8, 9.5e-8], n = 6	*β_i_* = [7.5e-9, 4.5e-8], n = 5; [5e-9, 2e-8] n = 6	*β_i_* = [5e-9, 1.75e-8], n = 5	*β_i_* = [3.3e-9, 1.16e-8], n = 5	*β_i_* = [2.5e-9, 8.7e-9], n = 5
		*β_d_* = [1e-6, 2.5e-5], n = 10; [2.5e-6, 4e-6], n = 5	*β_d_* = [0]; [7.5e-7, 7.75e-6], n = 5	*β_d_* = [0]; [7.5e-7, 7.75e-6], n = 5	*β_d_* = [0]; [7.5e-7, 7.75e-6], n = 5	*β_d_* = [0]; [7.5e-7, 7.75e-6], n = 5	*β_d_* = [0]; [7.5e-7, 7.75e-6], n = 5
Intermediate (ε = 0.0001)	0.01, 1, 10000	*β_i_* = NA	*β_i_* = [8e-8, 4e-7], n = 5	*β_i_* = [2e-8, 1e-7], n = 5	*β_i_* = [5e-9, 4.5e-8], n = 5	*β_i_* = [3.3e-9, 3.33e-8], n = 5	*β_i_* = [2.5e-9, 2.25e-8], n = 5
		*β_d_* = [1e-6, 1e-4], n = 10; [8e-6, 1.1e-5], n = 5	*β_d_* = [0]; [5e-7, 7.5e-6], n = 5	*β_d_* = [0]; [5e-7, 7.5e-6], n = 5	*β_d_* = [0]; [5e-7, 7.5e-6], n = 5	*β_d_* = [0]; [5e-7, 7.5e-6], n = 5	*β_d_* = [0]; [5e-7, 7.5e-6], n = 5
Frequency-dependent (ε = 1)	10000	*β_i_* = NA	*β_i_* = [2e-4, 1.7e-3], n = 5	*β_i_* = [5e-5, 4.2e-4], n = 5	*β_i_* = [3.1e-5, 1.56e-4], n = 5	*β_i_* = [2.1e-5, 1.04e-4], n = 5	*β_i_* = [1.6e-5, 7.85e-5], n = 5
		*β_d_* = [1e-2, 1.6e-1], n = 10; [4.5e-2, 6e-2], n = 5	*β_d_* = [0]; [1e-2, 1.224e-1], n = 5	*β_d_* = [0]; [1e-2, 1.224e-1], n = 5	*β_d_* = [0]; [1e-2, 1.224e-1], n = 5	*β_d_* = [0]; [1e-2, 1.224e-1], n = 5	*β_d_* = [0]; [1e-2, 1.224e-1], n = 5

Values are displayed as a range followed by the sampling intensity (n) employed equally over the range. Indirect transmission coefficients decreased in value and sampling range with increasing prion survival (*γ*), and increased in value and sampling range from density-dependent to frequency-dependent transmission.

### Plausible outcomes

Although there is substantial uncertainty surrounding many aspects of CWD transmission and dynamics, we do have some information on disease prevalence and host demographics from CWD epidemics in Wyoming, Colorado, and Wisconsin. Using the estimates of prevalence growth rates, peak apparent prevalence, and maximum host declines from the first 10 years of available data from monitored epidemics [20], [22], [26], we limited all of our possible simulation outcomes to ‘plausible’ results whose first 10 years of disease dynamics fell within certain boundaries established by the empirical data. We ignored data from captive studies because of the artificially high densities and resulting inflated contact rates. However, as there are many non-captive situations, including food-abundant agricultural fields, seasonal aggregations, and baiting stations that result in extremely high local densities of deer (up to 50 deer/km^2^ [58]) akin to those from pen studies, our metrics for defining ‘plausible’ outcomes may be considered conservative. We defined the beginning of our 10-year window as the first year in which prevalence ≥0.03, with the reasoning that this would be a reasonable threshold for detection in the field. Then, we defined a ‘plausible’ simulated epidemic as one that, within the first 10 years from detection experienced a peak prevalence ≤0.5, an annual prevalence growth rate, (prev_t = 10_/prev_t = 0_)^(1/10)^, less than or equal to 1.2 and an annual host population growth rate (*N*_t = 10_/*N*_t = 0_)^(1/10)^ greater than or equal to 0.95.

Among simulations run with the same direct and indirect transmission rates, there was very little stochastic variation in epidemic dynamics. Thus, for the purposes of summarizing and plotting a large amount of timeseries data, we calculated and plotted the average time series data, across multiple simulations, per unique combination of β's. Only active simulations, defined as runs where CWD successfully invaded the deer population (prev≥0.03), were included in these averages.

### R_0_ calculation

In order to estimate the impacts of prion survival on the basic reproductive number, *R*_0_, we used a modified version of our model to track the number of secondary infections caused by a single, infectious individual introduced to a completely susceptible population. We calculated this approximation of *R*_0_ by allowing only one individual to progress through all the disease stages. Other individuals within the population could become infected, but these infections were simply tallied and did not contribute to additional direct or indirect transmission events. The average number of secondary infections caused by a primary infectious individual (both through direct and indirect transmission events) from 10 simulations per unique combination of parameters served as our estimate of *R*_0_. This estimation method does not account for the interference of other infectious individuals, but did allow us to account for the continued births of new susceptible individuals that are likely to occur over the long timescales that prions may remain infectious in the soil. We allowed these simulations to run for 200 years to ensure that the environmental reservoir had completely decayed so as to be sure that we were not biasing our calculation of *R*_0_ low. We coded and ran all simulations using MATLAB® computing software (R2007a, The Mathworks, Inc.).

## Results

**Figure 3:**
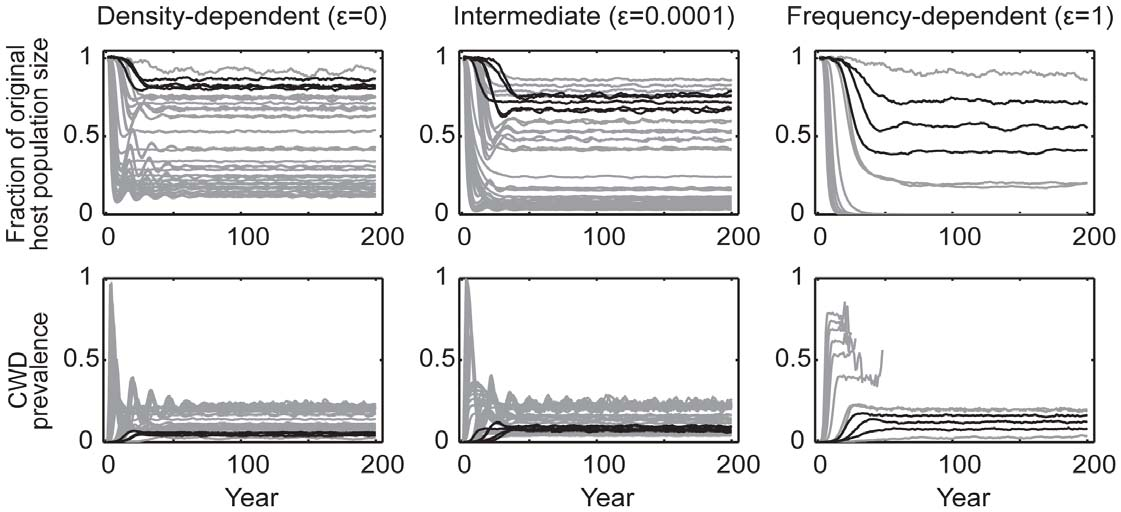
Simulations of CWD among 15,000 mule deer assuming only direct transmission. Host population dynamics (the fraction of the original host population size over time) and CWD prevalence are given assuming density-dependent (*ε* = 0), intermediate (*ε* = 0.0001), and frequency-dependent (*ε* = 1) transmission (columns 1, 2, and 3, respectively). Plots include aggregated (*k* = 0.01, 1) and non-aggregated (*k* = 10000) data. Lines represent the average of active simulations (mean = 8.8, sd = 1.9, range = 1–10) per direct transmission rate. Grey lines represent all possible outcomes, whereas black lines represent plausible outcomes, based on the epidemic characteristics observed in Colorado, Wyoming, and Wisconsin. None of the plausible simulation runs resulted in host extinction.

**Figure 4:**
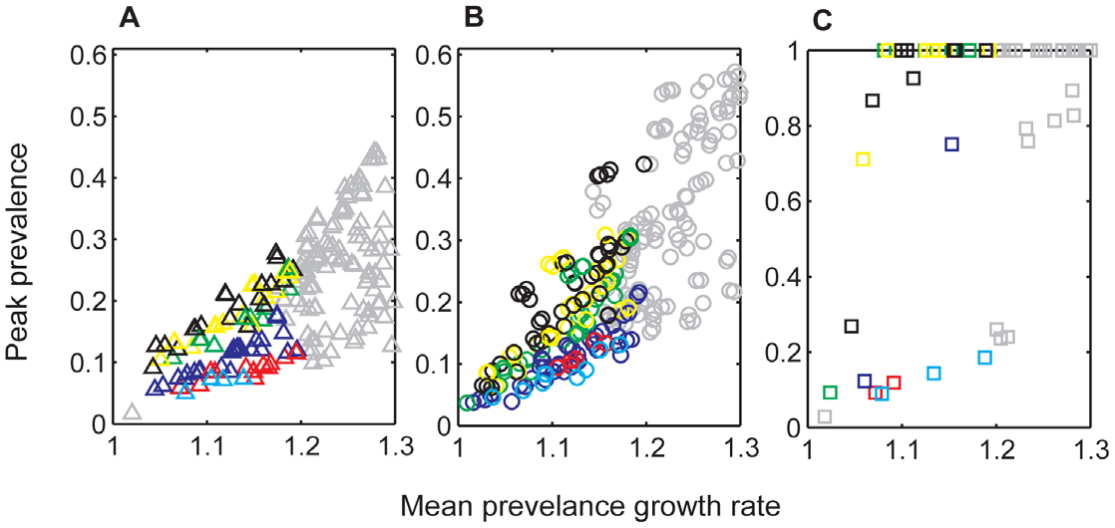
The relationship between peak prevalence and prevalence growth rate is modulated by prion persistence and the functional form of transmission. The mean prevalence growth rate calculated as the average growth rate during the first 10 years of the CWD epidemic, is plotted against the peak prevalence reached over the course of the entire CWD epidemic for (A) density-dependent (*ε* = 0), (B) intermediate (*ε* = 0.0001), and (C) frequency-dependent (*ε* = 1) transmission scenarios that assume both direct and indirect transmission. Plots include simulations with aggregated (*k* = 0.01, 1) and non-aggregated (*k* = 10000) transmission risk. Prion persistence is represented by color (cyan = direct transmission only, red = 3 month half life (HL), dark blue = 1 yr HL, green = 4 yr HL, yellow = 6 yr HL, black = 8 yr HL). Gray symbols represent model simulations that were not considered plausible given the epidemic characteristics observed in Colorado, Wyoming, and Wisconsin. Note the scale difference in the y-axis between plots A, B, and C. See Table 1 for *β_d_* and *β_i_* values employed in these simulations.

When we assumed that CWD is spread only through direct transmission, there were very few simulations that yielded plausible dynamics (Figure 3). Density-dependent (*ε* = 0) transmission generally produced oscillations in disease prevalence and host population size which dampened over time, converging on an endemic equilibrium (that is, if the initial oscillation did not result in host extinction) (Figure 3, column 1). We found that the initial prevalence growth rate and peak prevalence were tightly and positively correlated (Figure 4, cyan markers). Under direct, frequency-dependent transmission (*ε* = 1), the relationship between the initial prevalence growth rate and peak prevalence was variable, and host extinction was a much more probable outcome when compared to the other functional forms of transmission (Figure 3, column 3). Slightly frequency-dependent transmission (*ε* = 0.0001) yielded intermediate results where CWD prevalence often reached endemic equilibrium, but with greater propensities for more severe host population declines. Aggregation (*k* = 0.01 or 1) had the effect of dampening or eliminating oscillations in prevalence and host population size when we assumed transmission to be either density-dependent or weakly frequency-dependent (Figure S3).

**Figure 5:**
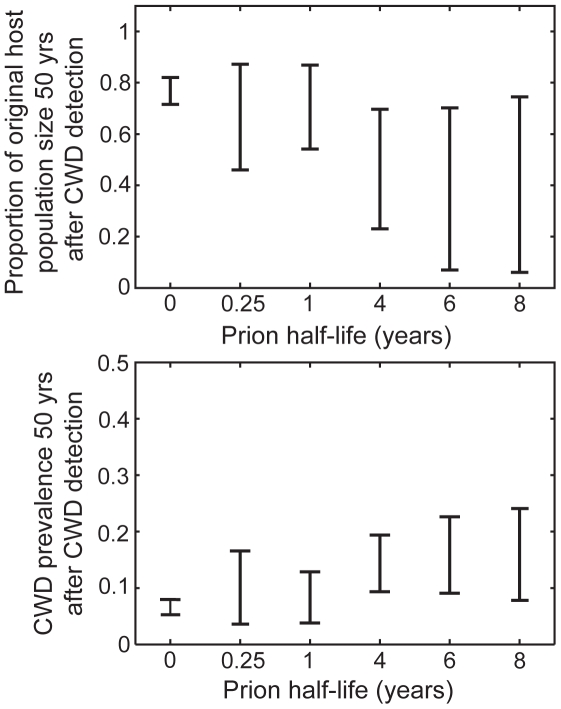
Impacts of prion half-life on host population size and disease prevalence. Ranges of plausible host population sizes (represented as the fraction of original host population size) (A) and disease prevalence (B), 50 years after the initial detection of CWD, as a function of prion half-life. Plots include results from simulations assuming density-dependent (*ε* = 0) and weakly frequency-dependent (*ε* = 0.001) indirect transmission, as well as aggregated (*k* = 0.01, 1) and non-aggregated (*k* = 10000) transmission risk (see Table 1 for *β_d_* and *β_i_* values). Results from simulations that assume only direct transmission (prion half-life = 0) are plotted for comparison. Plotted data represent only “plausible” outcomes which match the epidemic characteristics observed in the Colorado, Wyoming, and Wisconsin outbreaks. Ranges, rather than boxplots, are displayed since variation in sampling intensity may bias mean or median values.

**Figure 6:**
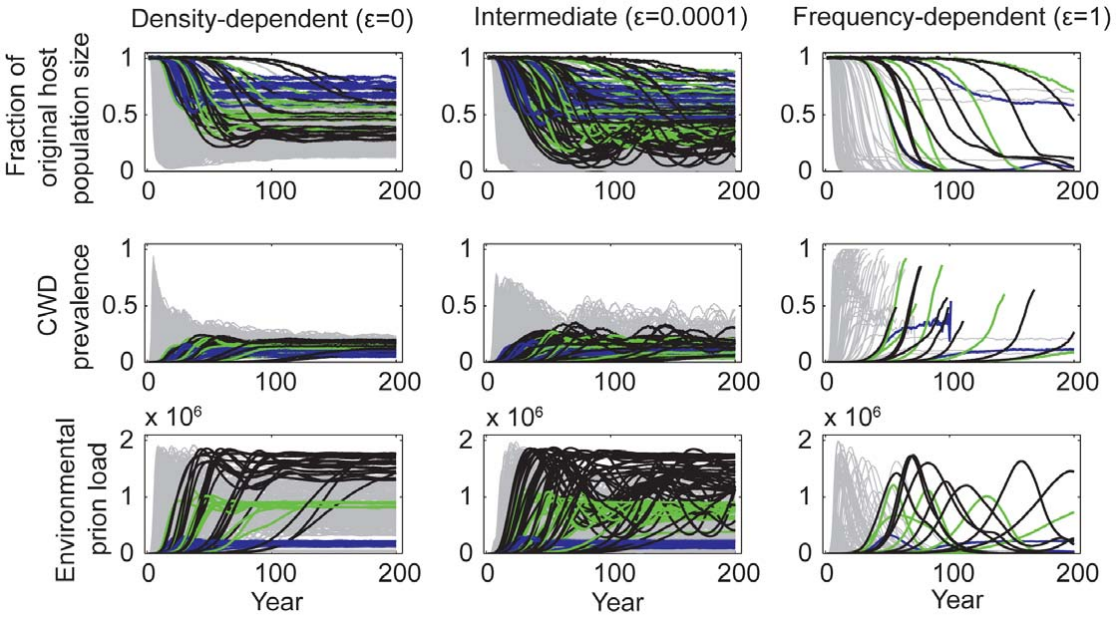
Dynamics of chronic wasting disease given a range of both direct and indirect transmission. Simulations of CWD among 15,000 mule deer were run assuming a wide range of direct and indirect transmission rates, density-dependent (*ε* = 0), intermediate (*ε* = 0.0001), and frequency-dependent (*ε* = 1) transmission (columns 1, 2, and 3, respectively), and a range of aggregation in infection risk (*k* = 0.01, 1, 10000; not distinguished in this figure). Host population dynamics, CWD prevalence, the environmental prion load, and the proportion of runs resulting in host extinction are displayed. Lines represent the average of active simulations (mean = 9.1, sd = 1.5, range = 2–10) per combination of direct and indirect transmission rates, prion survival, and aggregation. Grey lines represent all possible outcomes, whereas colored lines represent “plausible” outcomes assuming different prion survival rates (for ease of interpretation, only a subset of prion survival rates are presented: blue = 1 yr half-life (HL), green = 4 yr HL, and black = 8 yr HL). See Table 1 for *β_d_* and *β_i_* values employed in these simulations.

By contrast, indirect transmission yielded a wider range of disease dynamics that fell within the observed boundaries of the epidemics in Colorado, Wyoming, and Wisconsin. Although the general qualitative dynamics under the various functional forms of indirect transmission were similar to those from direct transmission models, the particular results were modulated by prion survival rates. For very low prion survival rates (half life = 0.25 yrs), prions never accumulated to appreciable levels in the environment. As a result, disease dynamics such as the long-term endemic prevalence and the degree to which hosts decline, mirrored those from direct transmission (Figure 5). However, as prion survival increased, so did the size of the environmental reservoir and the relative contribution of indirect transmission to overall disease dynamics. In a simple linear regression that included outputs from all model realizations across all of our explored parameter space, prion survival alone accounted for 29% and 31% of the variation in projected outcomes in CWD prevalence (R^2^ = 0.29, F = 82, df = 206, p<0.01) and the fraction of original host population remaining (R^2^ = 0.31, F = 96.8, df = 219, p<0.01) 50 years post-detection, respectively. Based on these simple regressions, we found that for every 1-year increase in prion half-life, prevalence 50 years post-detection is expected to increase 1 percentage point, (β = 9.3e-3, SE = 1.0e-3, t = 9.07, p<0.01; over the range of prion survival (0.45, 8 yrs) and CWD prevalence (3,35%)) and host populations are expected to decline an additional 4 percentage points (β = −4.3e-2, SE = 5.0e-3, t = −9.45, p<0.01; over the range of proportions of the original host population remaining (0,89%)) (Figure 5). Increasing prion survival, while holding everything else equal, increased the force of infection and the rate of epidemic growth. Thus ‘plausible’ simulation outcomes required relatively small indirect transmission rates (*β_i_*), resulting in patterns of longer delays to the onset of detectable prevalence and host mortality, longer periods of sustained epidemic growth, higher peak and endemic prevalences, and larger host declines for increasing values of prion survival (Figures 4 & 5). As seen in Figure 6, these patterns were largely driven by the dynamics of the environmental reservoir.

Simulations from all combinations of direct and indirect transmission rates, prion survival, and across various functional forms of transmission and aggregation, yielded a wide range of plausible (based on observed epidemics in Colorado, Wyoming, and Wisconsin), long-term outcomes for deer and CWD (Figure 6). Outcomes ranged from low endemic equilibria with small to moderate host declines, to sustained epidemics resulting in complete or functional host extinction (i.e. ≤10% of the original host population size). Host extinction was most probable when we assumed frequency dependent transmission (47% of runs went extinct within 200 years, with the majority of remaining runs on a trajectory for host extinction as well; Figure 6).

**Figure 7:**
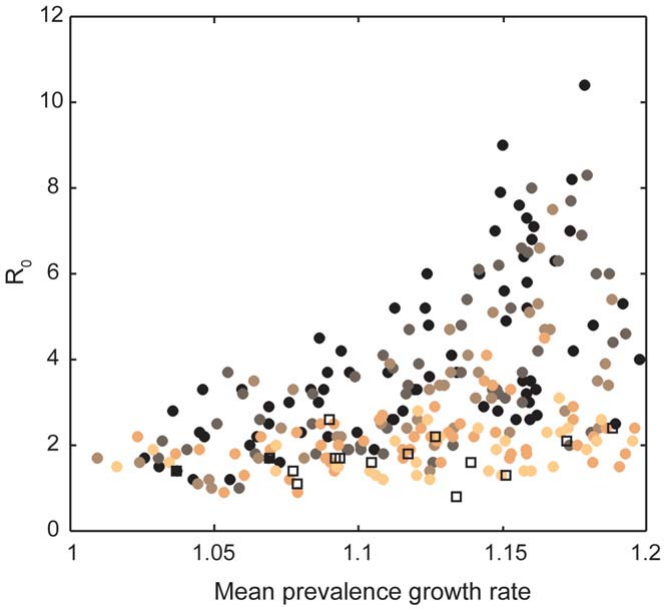
The basic reproductive number, *R*_0_, as a function of the prevalence growth rate and prion persistence. The mean prevalence growth rate is calculated as the average growth rate during the first 10 years of the CWD epidemic. Each point represents the average value for 10 simulations per unique combination of direct and indirect transmission rates, functional forms of transmission, aggregation, and prion persistence. Prion persistence ranges from low (lightest circles = 0.25 year half-life) to high (black circles = 8 year half-life). The empty squares represent values assuming only direct transmission. Plotted data represent only “plausible” simulation results. See Table 1 for *β_d_* and *β_i_* values employed in these simulations.

With the exception of frequency-dependent transmission for which there was no reliable relationship between prevalence growth rate and peak prevalence, increasing prion persistence increased the slope of the relationship between prevalence growth rate and peak prevalence (Figure 4). Similarly, although there was considerable variation due to relative rates of direct versus indirect transmission, we found that increasing prion survival rates generally increased *R*_0_ for a given prevalence growth rate (Figure 7; for every 1 year increase in prion half-life, we see an absolute increase of 0.29 in *R*_0_ [95%CI = 0.21–0.37; R^2^ = 0.21, F = 54.3, df = 202, p<0.01]). This suggests that *R*_0_ may actually be much larger than 1–2 despite observed prevalence growth rates of 1–1.2.

**Figure 8:**
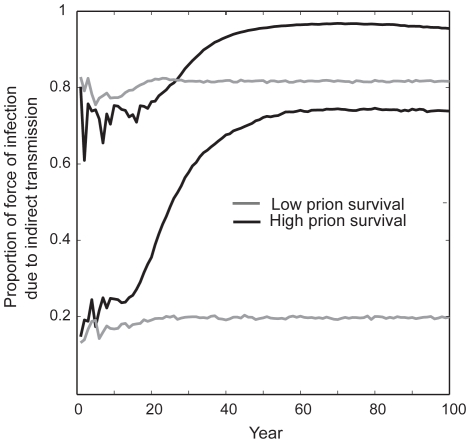
The proportion of the force of infection due to indirect transmission increases over time and varies with respect to prion survival. Gray lines represent typical dynamics when prion survival is low (0.25 year half-life), whereas black lines represent typical dynamics when prion survival is high (8 year half-life). The two lines plotted for each value of prion survival represent the cases where the starting ratio of indirect: direct transmission is either high (upper gray line, β*_d_* = 7.5e^−7^; β*_i_* = 5.75e^−8^; upper black line, β*_d_* = 2.25e^−6^; β*_i_* = 1.75e^−8^) or low (lower gray line, β*_d_* = 2.5e^−6^; β*_i_* = 1.1e^−8^; lower black line, β*_d_* = 7.5e^−6^; β*_i_* = 7.5e^−9^). Although not displayed here, all ranges of starting ratios are possible, but the patterns for low versus high prion survival remain the same.

Our results demonstrated how prion survival affects the relative contributions of direct versus indirect transmission to the per capita risk of infection over time. When prion survival was high, and the initial proportion of indirect transmission was low, we saw large increases in the proportion of risk due to environmental transmission over time as the environmental reservoir grew (Figure 8). By contrast, when prion survival was low, there was an initial increase in the proportion of infection risk due to environmental transmission, however, it quickly reached an equilibrium that mirrored the equilibrium in the environmental reservoir.

## Discussion

Many empirical and modeling studies of human and wildlife diseases have been conducted on directly-transmitted infections. Early work on these pathogens led us to believe that prevalence growth rates might linearly scale with peak prevalence and the effort required for disease control [1], [39]. However, insights gained from these systems may not apply to pathogens with long-lived environmental reservoirs such as anthrax, scrapie, and CWD. We found that if CWD prions remain infectious in the environment, epidemic metrics including prevalence growth rate, peak prevalence, and R_0_ are all expected to scale with the duration of prion persistence. In contrast to most studies that have modeled CWD transmission as being direct, our results suggest that despite relatively low prevalence growth rates, CWD epidemics fueled in part by indirect transmission may be characterized by higher values of *R*_0_, peak and endemic prevalences, and more severe host declines than previously thought.

The extremely slow and lethal dynamics of CWD have contributed both to the urgency and difficulty in predicting epidemic outcomes and effective control measures.

Unfortunately, we still struggle with uncertainty over the relative importance of direct versus indirect transmission, a distinction that we show has key consequences for disease dynamics, particularly if prions are environmentally long-lived. Most predictive modeling studies to date have made the a priori assumption that CWD is directly transmitted [27], [28], [29] (but see [59]); however, these studies struggle to capture both the comparatively low prevalence growth rates and the relatively high peak prevalences observed in Colorado or Wyoming's epidemics, for example. Here, we attempted to make few assumptions about the mode of CWD transmission and rather constrained our simulations to fit the first 10 years of observed epidemics in Colorado, Wyoming and Wisconsin. Using this approach, we demonstrated that there is a wider range of plausible host-pathogen outcomes than previously appreciated, the majority of which incorporate some portion of indirect transmission.

Several studies have attempted to use empirical data to differentiate between the relative contributions of direct versus indirect transmission of CWD under natural conditions. Miller et al. [32] used a statistical model-selection approach, and found greater model support for indirect transmission. Grear et al. [60] argue that the strong tendency for related females in Wisconsin to share disease status supports direct transmission of the disease, although they acknowledge that habitat use among closely related individuals is likely to be spatially correlated as well. We believe that a sharp distinction between direct and indirect transmission will often be impossible to decipher. Direct and indirect transmission lie along a continuum whereby direct transmission can be thought of as the limiting case where the pathogen has a very short half-life in the environment. For example, a deer contracting disease by licking another animal cannot be differentiated from a case where the individual ingests excreta very recently deposited by another animal. Only over longer timescales will disease dynamics differ between the two forms of transmission as the dynamics of the environmental pool of prions diverge from those of the host. These differences would be expected to increase as the environmental persistence becomes much longer than the host lifespan.

Indirect transmission and environmental prion persistence are problematic not only because they increase *R*_0_ (we found *R*_0_ values as high as 10–11, suggesting that initially, for every infected individual, we face having to control up to 10–11 new infections) and the probability of disease invasion and persistence [3], but also because we currently lack effective tools for mitigating the risk of indirect transmission. Disease control efforts based on reducing the density of individuals, and hence the number of infectious contacts, will not necessarily work with indirect transmission. Thus, the success of culling efforts at controlling the disease will depend on the proportion of transmission that is direct and density-dependent, as well as the proportion and timescale of indirect transmission. If prions are long-lived, the relative contribution of indirect transmission to the force of infection is likely to increase over time as the environmental reservoir grows. Reductions of infected hosts early in the epidemic should help limit the growth of the environmental reservoir and may offer the best opportunity for disease control (Figure 8).

The spatial response of deer to changing deer densities may also have important implications for the long term dynamics of CWD. We posit that the best-quality deer habitat, supporting the highest densities of deer, may be much more likely to become highly contaminated by CWD prions. This has the potential to turn the best habitat into ecological traps or sinks, whereby over time and through dispersal, deer are lured into prime habitat but then experience much higher infection and disease-induced mortality rates.

Epidemics of CWD in Colorado, Wyoming, and Wisconsin have yielded perplexing data regarding conflicting and changing age and sex-specific exposure patterns between the two endemic regions. Some of these differences may be due to differences in species' behavior, regional environments, or monitoring protocols. However, we posit that they may also stem from the relative contribution of indirect transmission, which is likely to increase over time during the epidemic (Figure 8). Factors driving indirect transmission such has habitat or landscape use, may differ from those that affect direct contacts such as host density and sex and age-specific behaviors. The epidemics in Colorado and Wyoming are much older than that in Wisconsin, and thus may currently be driven by a higher ratio of indirect to direct transmission. The differences in sex and age-specific risk both within and among sites may change as the pool of infectious environmental prions increases.

In addition to exploring the effects of indirect transmission, our study employed a larger range of functional forms of transmission than previous CWD studies, allowing for a greater range of plausible outcomes. Similar to Barlow [48], we found that incorporating aggregation in infection risk reduces the strong oscillations characteristic of density-dependent transmission, oscillations that we have not yet witnessed in any of the CWD endemic areas. We find it reassuring that aggregation, as a phenomenological proxy for modeling spatial heterogeneity, produces results qualitatively similar to models that explicitly incorporate space (e.g. reduced peak prevalences, slower prevalence growth rates, and the elimination of pronounced oscillations in prevalence and host numbers when compared to non-spatial model results) [61]. Although we cannot definitively say which form of transmission is a better representation of reality, transmission is likely to be somewhat dependent on host and prion density (so perhaps somewhere between 0<*ε*<1) and spatially aggregated [19], [24], [25]. Under these conditions, we found many plausible outcomes where CWD reaches equilibrium and only causes moderate population declines rather than complete host extinction. It is worth noting, however, that the only plausible simulations that captured the high prevalences of 35–50% reported in parts of WY (Figure 5), were those where we assumed a prion half-life of 8 years (Figure 4B), or those where we assumed frequency-dependent transmission (Figure 6), neither of which yield particularly hopeful prospects for host numbers or disease control.

Much investigation has demonstrated the remarkable stability of prions [62] and raising the question as to whether the concept of prion “half-life,” the ranges of values that we used, or the concept of a measurable *R*_0_, are reasonable. Despite the highly refractory nature of prions, it is likely that at least their bioavailability declines over time through natural, stochastic processes (e.g. prions may be covered over by soil/debris or are otherwise rendered unavailable to cervids). We do not know what this distribution of decay in bioavailability looks like (although it is likely to have a long right-tail), and our decision to model it as an exponential decay process was one of ease and convention rather than biological intuition [52], [53]. We did experiment with manipulating the shape of the survival distribution of prions in the environment, but aside from changing the timescales over which the disease dynamics played out (due to an increase or decrease in the rate at which the environmental reservoir accumulated), it did not affect our primary conclusions (data not shown). Furthermore, although prions may be capable of persisting much longer than that which we modeled (half-life>8 yrs) [38], based on the patterns in our simulations, we suspect that this would simply translate to even higher levels of disease prevalence, greater propensities for host declines, and even larger amounts of effort needed for disease control.

Our results highlight the uncertainty in the long-term prognosis for CWD and deer coexistence. Given the clear dependence of disease epidemic outcome on indirect transmission, improving our estimates of environmental prion survival and our ability to detect prions in the environment should increase our understanding of the disease and the effort needed for long-term CWD management and control. In the absence of such information, wildlife managers will continue to be faced with difficult decisions and limited confidence in model projections.

## Supporting Information

Figure S1 **Schematic representation of our stochastic susceptible(** ***S*** **)-exposed(** ***E*** **)-infectious(** ***I*** **)-clinical(** ***C*** **) simulation model for CWD in North American mule deer.** Duration of exposure, pre-clinical infectiousness, and the clinical state are modeled as log-normal distributions using the multi-compartmental ‘box-car’ approach (see Figure S2 for distributions). Plain, un-annotated arrows between *E*_1_∶*E*_15_ and *I*_1_∶*I*_23_ indicate a weekly transition probability = 1. Pacing individuals through these compartments ensures that they spend a minimum period of time in the exposed and infectious stages before transitioning to infectiousness or continuing as an infectious individual through to the clinical state and death. Remaining transitions are annotated with transition probabilities (*ρ*_1∶20_, *σ*_1∶21_, and *μ*_2∶36_; values reported in Table S1). Once in the infectious and clinical states, individuals shed (*τ*) and contribute prions upon death (*ϕ*) to the environmental reservoir, *V*, which in turn decays with a weekly probability of 1−*γ*.
(DOC)

Figure S2 **Distributions of the duration of exposure, infectiousness, and the clinical state for CWD in North American mule deer.** Means and ranges for the duration of exposure (mean = 27 weeks, range = 15–35 weeks), infectiousness (mean = 36 weeks, range = 25–44 weeks), and the clinical state (mean = 17 weeks, range = 1–36 weeks) were modeled after empirical and experimental data from deer. See Table S1 for transition probabilities that generate these distributions.
(DOC)

Figure S3 **Dynamics of CWD assuming high aggregation in infection risk and both direct and indirect transmission.** Host population dynamics, CWD prevalence, and the proportion of runs resulting in host extinction are given assuming density-dependent (*ε* = 0) and intermediate (*ε* = 0.0001) transmission (columns 1 and 2, respectively) and assuming high aggregation in infection risk (*k* = 0.01). Lines represent the average results from 10 simulations per combination of direct and indirect transmission rates. Grey lines represent all possible outcomes, whereas colored lines represent “plausible” outcomes assuming different prion survival rates (for ease of interpretation, only a subset of prion survival rates are presented: blue = 1 yr half-life (HL), green = 4 yr HL, and black = 8 yr HL). See Table 1 for *β_d_* and *β_i_* values employed in these simulations. Note that peak prevalence is reduced and oscillations in host population and CWD prevalence are dampened when compared to non-aggregated (and non-plausible) results displayed in Figure 6. None of the simulations resulted in host extinction.
(TIF)

Table S1 **Transition probabilities used to generate log-normal distributions of the duration of exposure, infectiousness, and the clinical phase.** The non-diseased mortality rate is denoted with an asterisk. See Figure S1 for a schematic of our model.
(DOC)
