# Supplementary material for: Modeling Routes of Chronic Wasting Disease Transmission: Environmental Prion Persistence Promotes Deer Population Decline and Extinction
Source: PLoS One. 2011 May 13;6(5):e19896. doi: 10.1371/journal.pone.0019896 (PMC3094393; doi:10.1371/journal.pone.0019896)
Supplement: Figure S1 — Schematic representation of our stochastic susceptible( S )-exposed( E )-infectious( I )-clinical( C ) simulation model for CWD in North American mule deer. Duration of exposure, pre-clinical infectiousness, and the clinical state are modeled as log-normal distributions using the multi-compartmental ‘box-car’ approach (see Figure S2 for distributions). Plain, un-annotated arrows between E 1∶E 15 and I 1∶I 23 indicate a weekly transition probability = 1. Pacing individuals through these compartments ensures that they spend a minimum period of time in the exposed and infectious stages before transitioning to infectiousness or continuing as an infectious individual through to the clinical state and death. Remaining transitions are annotated with transition probabilities (ρ 1∶20, σ 1∶21, and μ 2∶36; values reported in Table S1). Once in the infectious and clinical states, individuals shed (τ) and contribute prions upon death (ϕ) to the environmental reservoir, V, which in turn decays with a weekly probability of 1−γ. (DOC) [file pone.0019896.s001.doc]

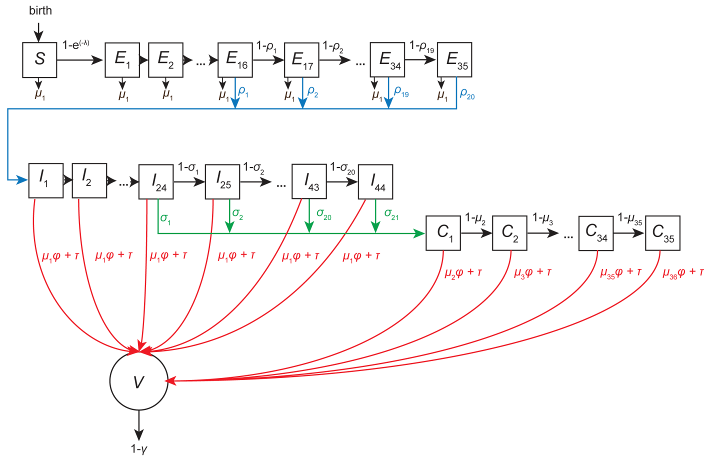


| **Symbol** | **Significance** |
| --- | --- |
| *S,E,I,C* | Susceptible, Exposed, Infectious, and Clinical states |
| *V* | Environmental reservoir of prions |
| *ρ*_1:20_ | Probability of transitioning from *E*_16_:*E*_35_ to *I*_1_ |
| *σ*_1:21_ | Probability of transitioning from *I*_24_:*I*_44_ to *C*_1_ |
| *μ*_1_ | Non-diseased probability of mortality |
| *μ*_2:36_ | Probability of mortality while in *C*_1_:*C*_36_ |
| *φ* | Per-capita contribution of prions at death |
| *τ* | Per-capita weekly rate of prion shedding |
| *γ* | Prion survival probability |
| *λ* | Force of infection |

**Figure S1. Schematic representation of our stochastic susceptible(*S*)-exposed(*E*)-infectious(*I*)-clinical(*C*) simulation model for CWD in North American mule deer.** Duration of exposure, pre-clinical infectiousness, and the clinical state are modeled as log-normal distributions using the multi-compartmental ‘box-car’ approach (see Figure S2 for distributions). Plain, un-annotated arrows between *E*_1_:*E*_15_ and *I*_1_:*I*_23_ indicate a weekly transition probability = 1. Pacing individuals through these compartments ensures that they spend a minimum period of time in the exposed and infectious stages before transitioning to infectiousness or continuing as an infectious individual through to the clinical state and death. Remaining transitions are annotated with transition probabilities (*ρ*_1:20_, *σ*_1:21_, and *μ*_2:36_; values reported in Table S1). Once in the infectious and clinical states, individuals shed (*τ*) and contribute prions upon death (*φ*) to the environmental reservoir, *V*, which in turn decays with a weekly probability of 1- *γ*.
