# Supplementary material for: Modeling Routes of Chronic Wasting Disease Transmission: Environmental Prion Persistence Promotes Deer Population Decline and Extinction
Source: PLoS One. 2011 May 13;6(5):e19896. doi: 10.1371/journal.pone.0019896 (PMC3094393; doi:10.1371/journal.pone.0019896)
Supplement: Figure S2 — Distributions of the duration of exposure, infectiousness, and the clinical state for CWD in North American mule deer. Means and ranges for the duration of exposure (mean = 27 weeks, range = 15–35 weeks), infectiousness (mean = 36 weeks, range = 25–44 weeks), and the clinical state (mean = 17 weeks, range = 1–36 weeks) were modeled after empirical and experimental data from deer. See Table S1 for transition probabilities that generate these distributions. (DOC) [file pone.0019896.s002.doc]

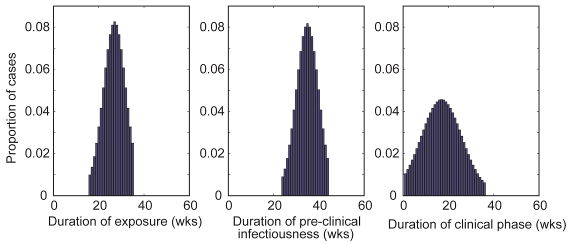


**Figure S2. Distributions of the duration of exposure, infectiousness, and the clinical state for CWD in North American mule deer.** Means and ranges for the duration of exposure (mean=27 weeks, range=15-35 weeks), infectiousness (mean=36 weeks, range=25-44 weeks), and the clinical state (mean=17 weeks, range=1-36 weeks) were modeled after empirical and experimental data from deer. See Table S1 for transition probabilities that generate these distributions.
