# Supplementary figures and images for: Modeling Routes of Chronic Wasting Disease Transmission: Environmental Prion Persistence Promotes Deer Population Decline and Extinction
Source: PLoS One. 2011 May 13;6(5):e19896. doi: 10.1371/journal.pone.0019896 (PMC3094393; doi:10.1371/journal.pone.0019896)

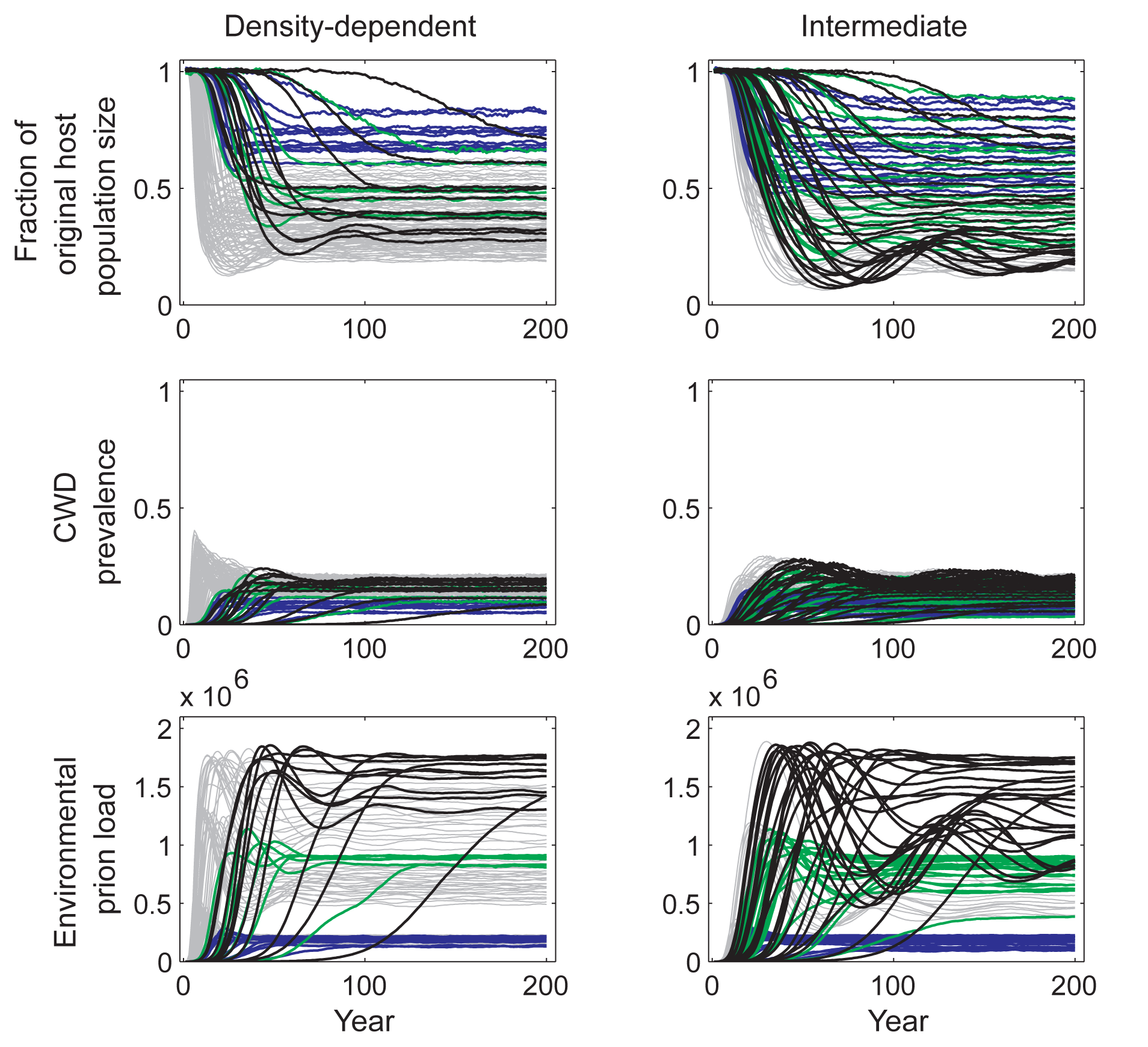

Supplement: Figure S3 — Dynamics of CWD assuming high aggregation in infection risk and both direct and indirect transmission. Host population dynamics, CWD prevalence, and the proportion of runs resulting in host extinction are given assuming density-dependent (ε = 0) and intermediate (ε = 0.0001) transmission (columns 1 and 2, respectively) and assuming high aggregation in infection risk (k = 0.01). Lines represent the average results from 10 simulations per combination of direct and indirect transmission rates. Grey lines represent all possible outcomes, whereas colored lines represent “plausible” outcomes assuming different prion survival rates (for ease of interpretation, only a subset of prion survival rates are presented: blue = 1 yr half-life (HL), green = 4 yr HL, and black = 8 yr HL). See Table 1 for βd and βi values employed in these simulations. Note that peak prevalence is reduced and oscillations in host population and CWD prevalence are dampened when compared to non-aggregated (and non-plausible) results displayed in Figure 6. None of the simulations resulted in host extinction. (TIF) [file pone.0019896.s003.tif]
