# Supplementary material for: Modeling Routes of Chronic Wasting Disease Transmission: Environmental Prion Persistence Promotes Deer Population Decline and Extinction
Source: PLoS One. 2011 May 13;6(5):e19896. doi: 10.1371/journal.pone.0019896 (PMC3094393; doi:10.1371/journal.pone.0019896)
Supplement: Table S1 — Transition probabilities used to generate log-normal distributions of the duration of exposure, infectiousness, and the clinical phase. The non-diseased mortality rate is denoted with an asterisk. See Figure S1 for a schematic of our model. (DOC) [file pone.0019896.s004.doc]

**Table S1. Transition probabilities used to generate log-normal distributions of the duration of exposure, infectiousness, and the clinical phase.** The non-diseased mortality rate is denoted with an asterisk. See Figure S1 for a schematic of our model.

| Subscript  index | Probability of transitioning from E_16_:E_35_ to I_1_ (ρ) | Probability of transitioning from I_24_:I_44_ to C_1_ (σ) | Non-diseased* and diseased mortality probabilities (governing transitions from C_1_:C_36_ to death) (μ) |
| --- | --- | --- | --- |
| 1 | 0.010 | 0.009 | 0.009* |
| 2 | 0.014 | 0.013 | 0.010 |
| 3 | 0.019 | 0.018 | 0.013 |
| 4 | 0.026 | 0.025 | 0.015 |
| 5 | 0.035 | 0.034 | 0.018 |
| 6 | 0.046 | 0.045 | 0.021 |
| 7 | 0.060 | 0.058 | 0.024 |
| 8 | 0.075 | 0.073 | 0.028 |
| 9 | 0.093 | 0.091 | 0.032 |
| 10 | 0.113 | 0.110 | 0.037 |
| 11 | 0.135 | 0.131 | 0.042 |
| 12 | 0.159 | 0.154 | 0.047 |
| 13 | 0.185 | 0.179 | 0.053 |
| 14 | 0.214 | 0.205 | 0.059 |
| 15 | 0.247 | 0.234 | 0.065 |
| 16 | 0.287 | 0.267 | 0.071 |
| 17 | 0.340 | 0.306 | 0.078 |
| 18 | 0.419 | 0.356 | 0.085 |
| 19 | 0.567 | 0.432 | 0.093 |
| 20 | 1 | 0.577 | 0.101 |
| 21 |  | 1 | 0.109 |
| 22 |  |  | 0.117 |
| 23 |  |  | 0.125 |
| 24 |  |  | 0.134 |
| 25 |  |  | 0.144 |
| 26 |  |  | 0.154 |
| 27 |  |  | 0.164 |
| 28 |  |  | 0.176 |
| 29 |  |  | 0.189 |
| 30 |  |  | 0.203 |
| 31 |  |  | 0.221 |
| 32 |  |  | 0.244 |
| 33 |  |  | 0.276 |
| 34 |  |  | 0.320 |
| 35 |  |  | 0.393 |
| 36 |  |  | 0.544 |
| 37 |  |  | 1 |
